# Btg2 is a Negative Regulator of Cardiomyocyte Hypertrophy through a Decrease in Cytosolic RNA

**DOI:** 10.1038/srep28592

**Published:** 2016-06-27

**Authors:** Yuki Masumura, Shuichiro Higo, Yoshihiro Asano, Hisakazu Kato, Yi Yan, Saki Ishino, Osamu Tsukamoto, Hidetaka Kioka, Takaharu Hayashi, Yasunori Shintani, Satoru Yamazaki, Tetsuo Minamino, Masafumi Kitakaze, Issei Komuro, Seiji Takashima, Yasushi Sakata

**Affiliations:** 1 Department of Cardiovascular Medicine, Osaka University Graduate School of Medicine, Suita, Osaka 565-0871, Japan; 2 Department of Medical Biochemistry, Osaka University Graduate School of Medicine, Suita, Osaka 565-0871, Japan; 3 Center of Medical Innovation and Translational Research, Osaka University Graduate School of Medicine, Suita, Osaka 565-0871, Japan; 4 Department of Cell Biology, National Cerebral and Cardiovascular Center, Suita, Osaka 565-8565, Japan; 5 Department of Cardiorenal and Cerebrovascular Medicine, Faculty of Medicine, Kagawa University, 1750-1 Ikenobe, Miki-cho, Kita-gun, Kagawa 761-0793, Japan; 6 Department of Clinical Medicine and Development, National Cerebral and Cardiovascular Center, Suita, Osaka 565-8565, Japan; 7 Department of Cardiovascular Medicine, Graduate School of Medicine, The University of Tokyo, Hongo, Bunkyo, Tokyo 113-8655, Japan

## Abstract

Under hypertrophic stimulation, cardiomyocytes enter a hypermetabolic state and accelerate biomass accumulation. Although the molecular pathways that regulate protein levels are well-studied, the functional implications of RNA accumulation and its regulatory mechanisms in cardiomyocytes remain elusive. Here, we have elucidated the quantitative kinetics of RNA in cardiomyocytes through single cell imaging and c-Myc (*Myc*)-mediated hypermetabolic analytical model using cultured cardiomyocytes. Nascent RNA labeling combined with single cell imaging demonstrated that Myc protein significantly increased the amount of global RNA production per cardiomyocyte. Chromatin immunoprecipitation with high-throughput sequencing clarified that overexpressed Myc bound to a specific set of genes and recruits RNA polymerase II. Among these genes, we identified *Btg2* as a novel target of Myc. Btg2 overexpression significantly reduced cardiomyocyte surface area. Conversely, shRNA-mediated knockdown of *Btg2* accelerated adrenergic stimulus-induced hypertrophy. Using mass spectrometry analysis, we determined that Btg2 binds a series of proteins that comprise mRNA deadenylation complexes. Intriguingly, Btg2 specifically suppresses cytosolic, but not nuclear, RNA levels. *Btg2* knockdown further enhances cytosolic RNA accumulation in cardiomyocytes under adrenergic stimulation, suggesting that Btg2 negatively regulates reactive hypertrophy by negatively regulating RNA accumulation. Our findings provide insight into the functional significance of the mechanisms regulating RNA levels in cardiomyocytes.

Cardiac hypertrophy is initially an adaptive response to exogenous stimuli caused by either high blood pressure or neurohumoral factors, and is recognized as the causative mechanism during the progression of heart failure. To adapt to the increased metabolic demand when they are subject to exogenous hypertrophic stimuli, cardiomyocytes accelerate RNA synthesis to promote translation and increase cellular protein synthesis^1^,^2^,^3^,^4^. Although the mechanisms for regulating protein levels, such as autophagy and ubiquitin-dependent proteasome degradation have been extensively studied^5^,^6^, the mechanism regulating RNA levels in cardiomyocytes remains to be elucidated.

c-Myc (*Myc*) is a major oncogene and is also a well-defined pro-hypertrophic transcription factor in cardiomyocytes^7^,^8^,^9^,^10^. Cardiomyocyte-specific depletion of Myc protein inhibits stress-induced cardiac hypertrophy and reactivation of the fetal gene program^11^. Conversely, Myc overexpression in cardiomyocytes induces cardiomyocyte hyperplasia during the developmental stage and cardiomyocyte hypertrophy in adult heart^9^,^12^,^13^,^14^. The molecular mechanisms underlying the pro-hypertrophic property of Myc are wide-ranging and have been linked to increased protein synthesis, reactivation of DNA synthesis, cell cycle reentry, mitochondrial biogenesis, and free fatty acid metabolism^11^,^12^,^13^,^14^. Of late, the regulation of global RNA production has been increasingly recognized as a key role of Myc. Ectopically overexpressed Myc either amplifies the output of existing gene expression program^15^,^16^ or accelerates transcription of specific target genes, leading to increased amounts of cellular RNA^17^,^18^. Although Myc is known to direct cardiomyocytes toward an anabolic state, the functional implications for RNA production and metabolism remain unclear. Furthermore, determining RNA kinetics in individual cardiomyocytes is challenging since previous studies measure cellular RNA amounts in populations of harvested cells.

A chemical method to detect RNA synthesis based on the biosynthetic incorporation of the uridine analog into newly transcribed RNA enables the quantitative evaluation of transcription rates in cultured cells^19^. We have used this nascent RNA labeling combined with single cell quantitative imaging to generate an analytical model based on cultured primary rat cardiomyocytes. Using this method, we detected the *Myc*-mediated hypermetabolic state in cardiomyocytes with accelerated RNA production per nucleus. From genome-wide analysis using chromatin immunoprecipitation followed by deep sequencing (ChIP-seq), we determined Myc-regulated genes in cardiomyocytes and identified *Btg2* as a novel Myc target gene. Based on biochemical and functional analysis using single cell imaging, we found that *Btg2* negatively regulates cardiomyocyte hypertrophy and decreases cytosolic RNA levels by interacting with the Ccr4-Not deadenylation complex.

## Results

### Increased amounts of Myc induce global RNA production in cardiomyocytes

**Figure 1:**
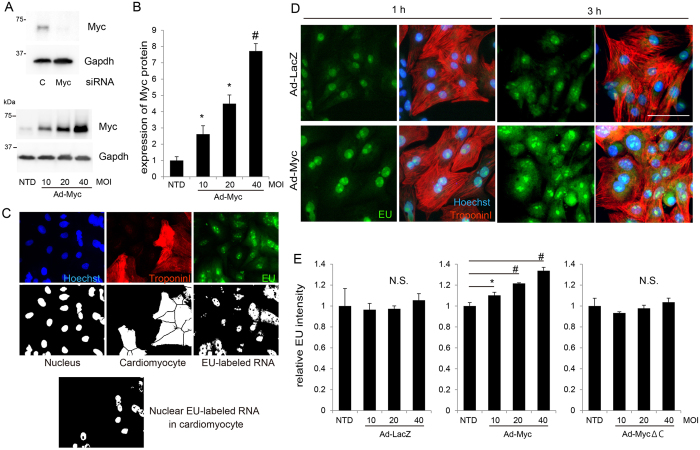
Myc induces global RNA production in cardiomyocytes. (**A**) Endogenous Myc protein in cardiomyocytes transfected with siRNA directed against *Myc* or with control siRNA (**C**) was detected by western blotting (upper panels). Adenovirally transduced *Myc* (Ad-Myc) in cardiomyocytes with increasing MOI (multiplicity of infection) was detected by western blotting (lower panels). Gapdh protein was used as a loading control. Before harvesting, cells were treated with 5 μM MG132 for 1 h. (**B**) Levels of overexpressed Myc protein in cardiomyocytes with increasing MOI normalized to Gapdh protein levels were calculated using imaging software (ImageQuantTL (GE), n = 3, mean ± SD). NTD: non transduction control. *p < 0.05, ^#^p < 0.01 vs NTD. (**C**) Algorithm for quantifying cardiomyocyte-specific nuclear EU-labeled RNA. First, cardiomyocytes were distinguished as troponin I–positive cells before their cell areas were segmented by locating the nuclei. EU images merged with nuclei specifically in cardiomyocytes were extracted and calculated as synthesized nuclear RNA per cardiomyocyte. Methodology details are shown in Fig. S1D. (**D**) Cardiomyocytes seeded in 96 well plates were transduced with either Ad-LacZ or Myc at MOI (multiplicity of infection) 20. Twenty-four hours after transduction, cells were labeled with EU for either 1 or 3 h before being fixed and immunostained. Representative images are shown. Scale bar: 50 μM. (**E**) Cardiomyocytes were transduced with either Ad-LacZ, Myc, or MycΔC at increasing MOI. Twenty-four hours after transduction, cells were labeled with EU for 1 h before being fixed and immunostained. Average nuclear EU intensities normalized to the cardiomyocyte cell number were calculated (n = 3, mean ± SD). *p < 0.05, ^#^p < 0.001 vs NTD.

A recent study concerning the role of Myc as a transcriptional amplifier determined cellular conditions with high Myc expression using ectopically induced Myc in lymphoma cells^15^. In our study, we transduced *Myc* (Ad-Myc) into a primary culture of rat neonatal cardiomyocytes using adenovirus to generate cellular conditions with increased amounts of Myc. We used a rabbit monoclonal antibody against Myc that specifically detected both endogenous and overexpressed Myc protein (Fig. 1A). Adenoviral transduction under gradually increased MOI resulted in cellular conditions with approximately 2 to 8 fold more Myc protein compared with its endogenous level (Fig. 1A,B). Transduction of Ad-Myc increased the level of *Npm1* mRNA, a well-known Myc target gene^20^, but Myc lacking the C-terminal DNA-binding domain (Ad-MycΔC)^21^ did not (Fig. S1A). To determine the effects of increased Myc levels on RNA production, we measured the total amount of synthesized RNA in cardiomyocytes using 5-ethnyluridine (EU) incorporation, which labels nascent transcribed RNA and is detected by click chemistry^19^. This technique was recently used to evaluate global RNA synthesis at the single cell level^22^. We observed EU-labeled RNA mainly in the nucleus, and to a lesser extent in the cytosol, of cardiomyocytes after 1 h of pulse labeling (Fig. S1B) as previously described^19^. To determine RNA synthesis in individual cardiomyocytes, we obtained immunofluorescence images of EU-labeled cardiomyocytes using an imaging cytometer. We applied a custom-made algorithm that can distinguish cardiomyocytes from non-cardiomyocytes as troponin I-positive cells, identify individual cell areas segmented by the location of the nucleus, and calculate the amount of synthesized nuclear RNA per cardiomyocyte (Fig. 1C, Fig. S1D,E). To determine the effect of Myc on RNA synthesis in cardiomyocytes, 24 h after transduction with either Ad-Myc or Ad-LacZ as a control, cardiomyocytes were labeled with EU for either 1 or 3 h. After labeling, cardiomyocytes were fixed and immunostained. As shown in Fig. 1D, Myc overexpression caused the amount of transcribed RNA in cardiomyocytes to increase significantly compared to the LacZ control. We calculated nuclear RNA production in cardiomyocytes as the average nuclear EU intensity normalized to the cardiomyocyte cell number using an imaging cytometer. We therefore determined that Ad-Myc induces a significant, dose-dependent increase in RNA synthesis in cardiomyocytes (Fig. 1E). In contrast, Ad-MycΔC overexpression did not exhibit the same effect despite the same overall amount of expression levels (Fig. S1C). These results demonstrate that the Myc protein induces an increase in global RNA synthesis in cardiomyocytes, and thus has a common function to amplify transcription across cell types^15^,^16^,^17^,^18^.

### Myc-binding induce RNA Pol II enrichment in cardiomyocytes

**Figure 2:**
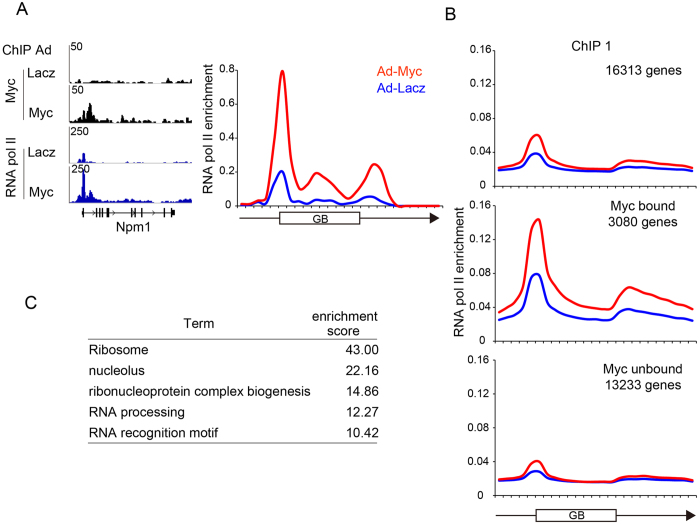
Myc-binding induce RNA Pol II enrichment in cardiomyocytes. (**A**) ChIP-seq mapped reads of Myc and Pol II at the *Npm1* locus (left) and the converted line graph for Pol II (right) following transduction with either Ad-LacZ or Myc. Vertical axis indicates the density of reads normalized to the total number of mapped reads, i.e., read density per million mapped reads per bp. (**B**) Average Pol II occupancy along the gene body at all 16,313 rat RefSeq genes (top), at the 3,080 genes preferentially bound by Myc (middle), or at the remaining 13,233 genes not bound by Myc (bottom). (**C**) Nine hundred forty-four Myc bound genes with the Pol II enrichment score that exhibited a greater than twofold increase in cardiomyocytes transduced with Ad-Myc compared with those transduced with Ad-lacZ control were subjected to DAVID functional annotation clustering analysis. Annotation terms for the top ranked categories with high enrichment scores are listed.

The transcribed RNA labeled by EU includes both protein-coding mRNAs transcribed by RNA polymerase II (Pol II) and non-coding RNAs transcribed by either RNA polymerase I or III^23^. To determine if a higher level of Myc increases the transcription of protein-coding mRNAs, and identify downstream targets of Myc in cardiomyocytes, we used ChIP-seq analysis to obtain genome-wide binding profiles both of Myc and Pol II. Twenty-four h after transducing cardiomyocytes with either Ad-Myc or Ad-LacZ as a control, the chromatin was extracted and subjected to ChIP analysis using monoclonal anti-Myc antibody and a monoclonal antibody specific for both phosphorylated and non-phosphorylated Pol II (8WG16)^18^. Peak calling was performed using MACS (Model-based Analysis for ChIP-Seq)^24^ on both ChIP and input samples, and we identified a total of 3,080 genes preferentially bound by Myc in cardiomyocytes under the conditions with increased amount of Myc. In contrast, in cardiomyocytes transduced with the *LacZ* control, only 94 genes were detected, probably due to the extremely low level of endogenous Myc protein present in cardiomyocytes under basal conditions. The Pol II peaks were quantified according to the literature^25^ with modifications using WIG-format files (Fig. S2A, analytical details are in Materials and Methods). Briefly, we divided the gene body defined as the entire gene from the transcription start site to the end of the transcript^26^ into 10 blocks. Pol II occupancy in each block from 5 blocks in the upstream region to 10 blocks in the downstream region (a total of 25 blocks) was calculated as the integrated values of the peaks. Each quantified value was normalized to the gene length for relative comparison, since the absolute amount of Pol II enrichment calculated from mapped reads along the genome depends on the gene length. As an example, Myc bound the promoter region of *Npm1* and recruited Pol II to both the promoter and gene body when compared to LacZ control (Fig. 2A). For other genes previously determined as Myc-targeted genes, Myc was found to bind directly to these genes and recruited Pol II in the replicated experiments (Fig. S2B). To evaluate the global effect of *Myc* transduction in rat cardiomyocytes, we calculated the average Pol II enrichment at all 16,313 rat RefSeq genes. As shown in Figs 2B and S2D (replicated experiments), overexpressed Myc significantly induced Pol II recruitment along the gene body. At the 3,080 genes preferentially bound by Myc, Pol II recruitment following *Myc* transduction was more prominent, whereas Pol II recruitment at the 13,233 Myc-unbound genes were modest. Interestingly, at the 3,080 Myc-targeted genes, pre-accumulation of Pol II was observed under basal conditions (*LacZ* transduction), indicating that the amplification of active gene expression by Myc^15^,^16^ is the common molecular property even in primary cultured cardiomyocytes. In contrast, we found that overexpressed Myc bound both the promoter region and body of many cardiac-specific genes including transcription factors (*Gata4*, *Gata6*, *Tbx20*, *Nkx2-5*), structural genes (*Actc1*, *Pln*), or heart failure related genes (*Nppa*, *Nppb*), but did not induce Pol II recruitment (Fig. S3A). We further verified that the mRNA levels of heart failure–related genes (*Nppa, Nppb*, and *Acta1*) in cardiomyocytes transduced with Myc both under serum-supplemented conditions and serum-free conditions. As shown in Fig. S3B, overexpression of Myc did not increase the levels of either *Nppa*, *Nppb*, *or Acta1* mRNA, suggesting that Myc alone is not sufficient to upregulate expression of these genes. These data are consistent with a previous finding that overexpressed Myc invades accessible chromatin in lymphocytes^17^ and that Myc binding does not necessarily upregulate transcription in murine fibroblasts^27^. To distinguish the major downstream effector genes induced by Myc in cardiomyocytes, we calculated the Pol II enrichment score by summing the normalized values for Pol II peaks along the gene body of each gene (Fig. S2A right), and identified 944 genes for which Pol II recruitment caused a greater than 1.5-fold increase in cardiomyocytes transduced with Ad-Myc compared with those transduced with Ad-LacZ control (Table S1). To validate the Pol II enrichment score, the mRNA expression levels of the 25 highest-ranked genes according to the ratio of the Pol II enrichment score (Ad-Myc/Ad-LacZ) were determined by quantitative real-time PCR. As shown in Fig. S4A,S4B, the mRNA levels of almost all the selected genes were significantly higher in cardiomyocytes transduced with Ad-Myc. The 944 genes with increased Pol II enrichment score were subjected to functional enrichment analysis using the software DAVID^28^. The annotation terms for these genes associated with both Myc binding and Pol II recruitment are significantly enriched in ribosome, nucleolus, ribonucleoprotein complex biogenesis, RNA processing, and RNA recognition motif (Fig. 2C), which are defined as Myc-targeted genes in other cell types^27^,^29^. The detailed annotation terms and categories of the genes with (944 genes) or without (2136 genes) pol II enrichment are shown in Table S2. These data suggest that the increased amount of Myc results in significant Pol II enrichment to the targeted genes in cardiomyocytes, and also that Myc preferentially and directly targets genes involved in ribosomal biogenesis and RNA metabolism in cardiomyocytes.

### Myc targets *Btg2*, which is specifically and transiently upregulated in cardiomyocytes under adrenergic stimulation

**Figure 3:**
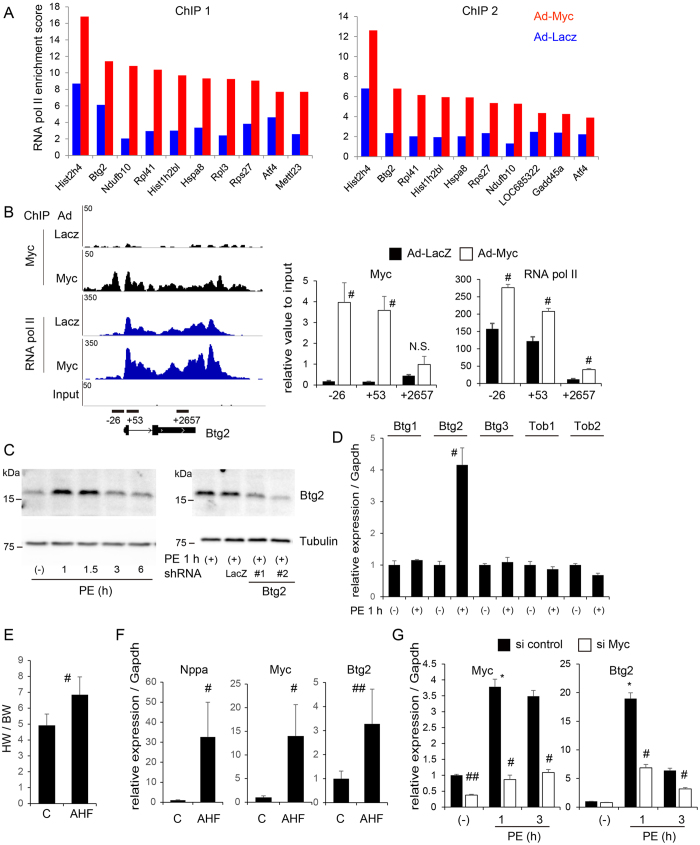
Myc targets *Btg2*, which is transiently upregulated in cardiomyocytes under adrenergic stimulation. (**A**) Highly ranked genes in quantified RNA Pol II enrichment score under Ad-Myc transduction conditions in the replicated experiments. (**B**) Mapped reads for Myc and Pol II at the *Btg2* locus and the primer locations used for the quantitative real-time PCR. The numbers at X-axis indicate the relative genomic location (bp) to the *Btg2* transcription start site (left). Quantitative real-time PCR evaluation of Myc and Pol II binding at the *Btg2* locus in ChIP analysis (right, n = 3, mean ± SD). ^#^p < 0.001 vs Ad-LacZ. (**C**) Primary cultured rat neonatal cardiomyocytes were starved for 12 h and treated with PE for the indicated time (left). Cardiomyocytes were transfected with shRNA directed against either *LacZ* or *Btg2* (#1 and #2). Twenty-four hours after transduction, cells were starved for 12 h, then treated by PE for 1 h (right). Endogenous Btg2 in cardiomyocytes at each condition was detected by western blotting. Tubulin was used as a loading control. (**D**) mRNA levels of *BTG*/*TOB* family genes were determined by quantitative real-time PCR in cardiomyocytes treated with PE for 1 h (n = 3, mean ± SD). ^#^p < 0.01. (**E**) Acute pressure overload was induced by transaortic constriction (TAC) in murine hearts. Forty-eight hours after TAC, heart tissues were isolated and the heart weight (HW) normalized to body weight (BW) was measured (n = 4 each, mean ± SD). ^#^p < 0.01. (**F**) mRNA expression levels for indicated genes in ventricular heart tissues isolated as in (**E**) were evaluated by quantitative real-time PCR (n = 4 each, mean ± SD). ^#^p < 0.01. ^##^p < 0.05. (**G**) Cardiomyocytes were transfected with siRNA directed against *Myc* or with control siRNA. Twenty-four hours after transfection, cardiomyocytes were starved for 12 h, and treated with PE for the indicated time. *Myc* and *Btg2* mRNA levels at each time point were determined by quantitative real-time PCR (n = 3, mean ± SD). *p < 0.01 vs no treatment with si control. ^#^p < 0.05, ^##^p < 0.01 vs si control.

We ranked Myc-bound genes according to their Pol II enrichment score at the gene body that was normalized to gene length in cardiomyocytes transduced with Myc (Fig. 3A). The ratio of the Pol II enrichment score and the change in mRNA levels are shown in Fig. S4C,S4D. From the replicated analyses, the high ranked genes included histone protein, ribosomal protein, and heat shock protein that were previously determined as Myc target genes. From among these genes, we identified B-cell Translocation Gene 2 (*Btg2*, also known as *Tis21* and *Pc3*) as ranking second in Pol II enrichment in both experimental replicates. Myc and Pol II bindings at the *Btg2* locus were confirmed by quantitative PCR using specific primer sets (Fig. 3B). Btg2 is a BTG/TOB family protein that contains a conserved BTG domain. *BTG*/*TOB* family genes are antiproliferative genes that participate in various biological processes^30^,^31^. *Btg2* was first isolated in screens designed to identify primary response genes induced by growth factors in PC12 cells or mitogenic agent in 3T3 cells^32^,^33^. First, we determined the levels of endogenous Btg2 protein in cardiomyocytes under hypertrophic stimuli using phenylephrine (PE), an α1-adrenergic receptor agonist. As shown in Fig.3C, Btg2 expression levels were induced immediately and transiently following the addition of PE, with expression reaching maximum levels within 1 h of PE addition (Fig. 3C, left). Western blot analysis determined that Btg2 protein levels were decreased by transfection with either of two shRNA against *Btg2* (Fig. 3C, right). *Btg2* transcript levels were immediately induced in cardiomyocytes after 1 h PE treatment, and specifically, other *BTG*/*TOB* family genes were not induced under the same conditions (Fig. 3D). To determine whether *Btg2* is functionally relevant to the pathological hypertrophic response in cardiomyocytes, we prepared pressure-overloaded murine ventricular heart tissues by transverse aortic constriction (TAC) that induced significant cardiac hypertrophy (Fig. 3E). Forty-eight hours after TAC, *Btg2*, *Myc*, and *Nppa* mRNA levels in ventricular heart tissues were significantly upregulated (Fig. 3F). Next, to determine whether the immediate induction of *Btg2* is mediated by Myc, we knocked down *Myc* expression using siRNA in cardiomyocytes. As shown in Fig. 3G, suppression of Myc protein prevented the upregulation of *Btg2* expression in PE-treated cardiomyocytes, albeit not completely. Combined with findings that Myc binds directly to the *Btg2* locus and induces Pol II recruitment, these data suggest that *Btg2* is immediately and transiently induced in cardiomyocytes under adrenergic stimulation and that Myc is required for the induction of *Btg2* at least partly in cardiomyocytes.

### Btg2 represses cardiomyocyte hypertrophy by interacting with the Ccr4–Not deadenylation complex

**Figure 4:**
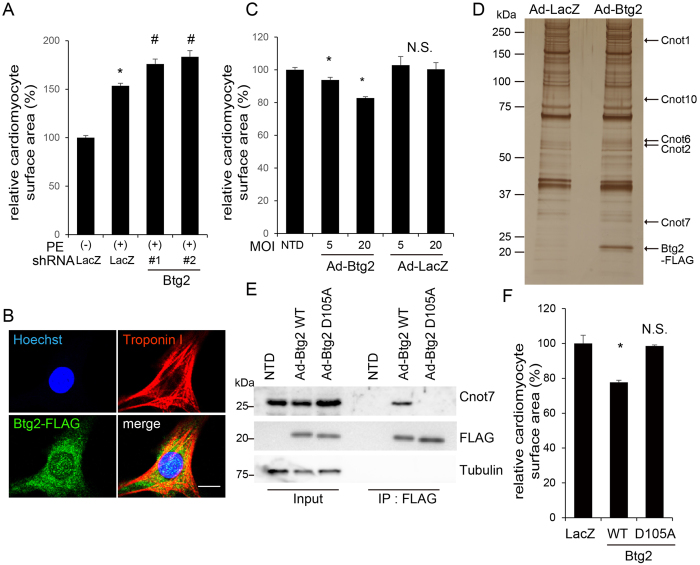
Btg2 represses cardiomyocyte hypertrophy by interacting with the Ccr4–Not deadenylation complex. (**A**) Cardiomyocytes seeded in 96 well plates were transduced with shRNA against either *LacZ* or *Btg2*. Twenty-four hours after transduction, cardiomyocytes were starved for 12 h and treated with PE for 12 h before they were fixed and immunostained. Fluorescence images were obtained with an imaging cytometer. The average cardiomyocyte surface areas were calculated and normalized by LacZ control without PE (n = 3, mean ± SD). *p < 0.0001 vs without PE, ^#^p < 0.001 vs LacZ control with PE. (**B**) Cardiomyocytes transduced with adenovirus encoding C-terminally FLAG-tagged Btg2 were fixed and immunostained using anti FLAG and anti troponin I antibodies. Scale bar: 10 μM. (**C**) Cardiomyocytes were transduced with Ad-Btg2 or LacZ at increasing MOI. Twenty-four hours after transduction, cell surface areas were calculated as in (**A**) (n = 3, mean ± SD). *p < 0.001 vs NTD (non-transduction). (**D**) Cardiomyocytes were transduced with Ad-LacZ or Ad-Btg2. Forty-eight h after transduction, total cell lysates were immunoprecipitated using anti-FLAG antibody. Eluted samples were subjected to mass spectrometry analysis. The same samples were resolved by SDS-PAGE and silver-staining. Representative bound proteins detected by mass spectrometry analysis are indicated. (**E**) Cardiomyocytes were treated as in (**D**). The immunoprecipitated samples and inputs were resolved by SDS-PAGE and subjected to western blotting using the indicated antibodies. Tubulin was used as a loading control. (**F**) Cardiomyocytes seeded in 96 well plates were transduced with either Ad-LacZ, Ad-Btg2 WT or Ad-Btg2^D105A^ at MOI 20. Twenty-four hours after transduction cell surface areas were calculated as in (**A**) (n = 3, mean ± SD). *p < 0.001 vs LacZ.

To determine the physiological role of *Btg2* during the hypertrophic response in cardiomyocytes, we knocked down *Btg2* expression using shRNA in cardiomyocytes stimulated by PE. Both before and after PE treatment, cardiomyocyte surface area detected by troponin I staining was calculated using an imaging cytometer (Fig. S5A). As shown in Figs 4A and S5B, *Btg2* knockdown using two different shRNA significantly promoted cardiomyocyte hypertrophy induced by PE treatment. We generated adenovirus encoding C-terminally FLAG-tagged Btg2 protein (Ad-Btg2) and transduced it in cardiomyocytes. Overexpressed Btg2 was diffusely distributed mainly in the cytosol and partially in the nucleus in cardiomyocyte (Fig. 4B). As shown in Figs 4C and S5C, overexpressed Btg2 significantly decreased the size of cardiomyocytes in a dose-dependent fashion. *Btg2* belongs to antiproliferative *BTG*/*TOB* gene family and has diverse functions^30^,^31^,^34^ as a transcriptional co-regulator^35^,^36^,^37^, a regulator of embryonic development^38^,^39^, a tumor suppressor^40^,^41^,^42^, a regulator of the cell cycle^43^,^44^, and in mRNA deadenylation and decay^45^,^46^. To determine the major downstream mechanism underlying the *Btg2*-mediated suppression of cardiomyocyte hypertrophy, we performed a screen for Btg2 binding partners in cardiomyocytes. Btg2-FLAG overexpressed in cardiomyocytes was immunoprecipitated and the eluted sample was subjected to mass spectrometry analysis. As shown in Fig. 4D, Fig. S5D, and Table S3, Btg2 bound preferentially to a series of proteins that comprise the Ccr4–Not deadenylation complex. Protein–protein interactions between Btg2 and Cnot7, a catalytic subunit of the Ccr4–Not complex^46^,^47^,^48^, were confirmed by immunoprecipitation followed by western blotting (Fig. 4E). The Btg2 mutant harboring the D105A amino acid substitution, which inhibits the interaction between Btg2 and Cnot7^49^,^50^, did not bind Cnot7 in cardiomyocytes (Fig. 4E). Importantly, Btg2^D105A^ did not reduce cardiomyocyte surface area compared with cells expressing a similar amount of wild-type Btg2 (Fig. 4F and Fig. S5E), suggesting that the interaction with the Ccr4-Not complex is required for the inhibitory effect of Btg2 observed in cardiomyocytes. Although the previously identified downstream molecular pathways of *Btg2* are wide-ranging, our data, including MS spectrometry analysis, suggest that the major downstream effectors in cardiomyocytes are involved in RNA degradation pathways.

### Btg2 acts as a counterpart to pro-hypertrophic stimuli by reducing cytosolic RNA levels

**Figure 5:**
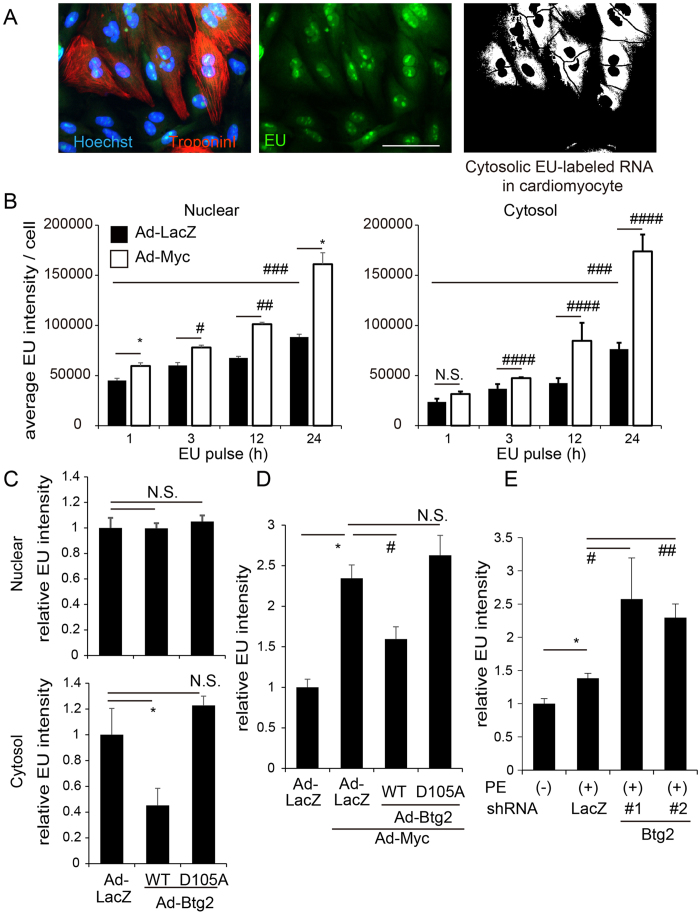
Btg2 acts as a counterpart to pro-hypertrophic stimuli by reducing cytosolic RNA levels. (**A**) The quantification algorithm was modified to evaluate cytosolic EU intensity in cardiomyocytes. In addition to the previous protocol, nuclear imaging results were subtracted from entire EU images in cardiomyocytes. The cytosolic EU intensities were specifically extracted thereafter (right). Scale bar: 50 μM. Methodological details are shown in Fig. S1F. (**B**) Cardiomyocytes were transduced with either Ad-LacZ or Ad-Myc. Twenty-four hours after transduction, the cells were labeled with EU for the indicated time before they were fixed and immunostained. Nuclear and cytosol EU intensities were differentially quantified (n = 3, mean ± SD). *p < 0.005, ^#^p < 0.01, ^##^p < 0.001, ^####^p < 0.05 vs Ad-LacZ. ^###^p < 0.0001 analyzed by one way ANOVA with repeated measures vs Ad-LacZ results. (**C**) Cardiomyocytes were transduced with either Ad-LacZ, Ad-Btg2 WT, or Ad-Btg2^D105A^. Twenty-four hours after transduction, cells were labeled by EU for 24 h before they were fixed and immunostained. Nuclear and cytosol EU intensities were differentially quantified by imaging cytometer (n = 3, mean ± SD). *p < 0.05 vs Ad-LacZ. (**D**) Cardiomyocytes were either transduced with Ad-LacZ or co-transduced with Ad-Myc and either Ad-LacZ, Ad-Btg2 WT, or Ad-Btg2^D105A^. Twenty-four hours after transduction, cells were labeled with EU for 24 h before they were fixed and immunostained. Cytosolic EU intensity was quantified as in (**B**) (n = 3, mean ± SD). *p < 0.001 vs Ad-LacZ. ^#^p < 0.05 vs Ad-LacZ. (**E**) Cardiomyocytes were transduced with shRNA against *LacZ* or *Btg2* (#1 and #2). Twenty-four hours after transduction, cells were starved for 12 h, treated by PE, and simultaneously labeled with EU for 24 h. The cells were subsequently fixed and immunostained. Cytosolic EU intensity was calculated (n = 3, mean ± SD). *p < 0.005 vs without PE. ^#^p < 0.05, ^##^p < 0.005 vs LacZ.

Previous findings suggest that Btg2 affects the deadenylase activity of Ccr4-Not complex^30^,^46^ based on determination of the crystal structures of Btg2 and Ccr4-Not subunits^49^, the functional analysis of cell proliferation^50^, or on biochemical analysis that identified Btg2 as a general activator of mRNA deadenylation through the Ccr4–Not complex^45^. Major RNA decay machineries, including the Ccr4–Not complex, are cytoplasmic^51^. Therefore, detailed assessment targeting regional measurement of RNA levels further supports the functional relationship between Btg2 and RNA degradation pathways. We modified our analytical algorithm to measure the cytosolic RNA accumulation in individual cardiomyocytes (Fig. 5A and Fig. S1D). We compared the different EU pulse labeling times to detect cytosolic RNA, and both the average nuclear and cytosolic EU intensities per cell increased in a time-dependent manner in cardiomyocytes (Fig. 5B) as previously reported^19^. Under these analytical conditions, Ad-Myc transduction not only accelerated nuclear RNA production but also increased cytosolic RNA accumulation (Fig. 5B), suggesting that accelerated RNA synthesis induced by overexpressed Myc exceeded the processing capacity of cardiomyocytes. Since the extension of labeling time more than 24 h increased cell toxicity, we used a 24 h labeling time for further analysis. Intriguingly, Btg2 overexpression significantly repressed the amount of cytosolic RNA but did not affect the amount of nuclear RNA (Fig. 5C). Furthermore, co-transduction of Ad-Btg2 inhibited cytosolic RNA accumulation induced by overexpressed Myc (Fig. 5D) and decreased the size of the cardiomyocytes (Fig. S5F). These suppressive effects on cytosolic RNA production were not observed in cells transduced with Ad-Btg2 D105A. To further confirm whether the effects of Btg2 on the decrease of cytosolic RNA are mediated through the Ccr4-Not complex, *Cnot7* was knocked down using shRNA in cardiomyocytes. As shown in Fig. S5G and S5H, the decreased cytosolic EU intensity caused by Ad-Btg2 transduction was significantly rescued by *Cnot7* knockdown. Finally, we determined the cytosolic RNA amounts under physiological stimulation mediated by PE in cardiomyocytes. As shown in Fig. 5E, cytosolic RNA was significantly increased under PE stimulation and further accumulated when *Btg2* was knocked down in cardiomyocytes. Our single cell quantitative imaging analysis of cardiomyocytes found that Btg2 negatively regulates cardiac hypertrophy, opposing pro-hypertrophic stimuli by modulating cytosolic RNA levels.

## Discussion

In this study, we used single cell quantitative imaging combined with nascent RNA labeling to elucidate the functional significance of the mechanisms that regulate RNA levels in cardiomyocytes. In our analytical model, Myc promoted nuclear RNA synthesis in cardiomyocytes in a dose-dependent fashion, thereby amplifying transcription. From the genome-wide binding profiles for Myc and Pol II using ChIP-seq, we determined that Myc binds preferentially to and recruits Pol II to a specific set of genes that includes genes involved in ribosomal and RNA biogenesis. This finding suggests that even in primary cardiomyocytes, Myc targets genes involved in biomass accumulation, which has been reported as a primordial function of Myc in tumor cells^29^. In cardiomyocytes, Myc binds directly not only to the above targets but also to cardiac-specific genes. Interestingly, Myc binding did not induce Pol II recruitment to genes involved in the hypertrophic response (*Actc1*, *Nppa*, *Nppb*) that are transcriptionally upregulated under adrenergic stimulation or pressure-overloaded heart failure. These data suggest that Myc binding is not sufficient to promote transcription of hypertrophy-related genes, although Myc is required to activate these genes^11^. Myc binding at these genes may be mediated by open chromatin invasions by excess Myc^15^,^17^, which is a common molecular phenomenon across cell types. The general transcription factor TFIIB plays a central role in preinitiation complex assembly, providing a bridge between promoter-bound TFIID and RNA polymerase II^52^. Furthermore, TFIIB has a pivotal role in regulating gene transcription during the progression of cardiomyocyte hypertrophy^53^. Since overexpression of Myc recruits pol II to the specific set of genes in rat neonatal cardiomyocytes, it is likely that pol II recruitment in our experimental model is mediated by TFIIB.

RNA pol II dynamics, distribution and regulation in cardiac myocyte hypertrophy have been reported^53^,^54^,^55^. An exploratory approach using quantified Pol II enrichment score in our experiments identified *Btg2* as a novel Myc target gene. Immediate early genes with extremely short half-lives like *Btg2* may be difficult to detect by expression profiling because mRNA expression levels depend on the amount of mRNA at the time analyzed. In contrast, Pol II accumulation at a specific gene locus might indicate the transcriptional signature including the past time. Therefore, molecular screening using quantified Pol II signatures is useful to identify downstream targets with very short half-lives regulated by specific transcription factors. These data are consistent with previous findings that Pol II occupancy detected by ChIP-seq provides a precise readout for transcription and is useful for detecting genes that have high mRNA turnover rates^56^. Although *Btg2* is rapidly induced by pro-hypertrophic stimuli in cardiomyocytes, its physiological role appears to be that of a negative regulator that opposes anabolism. It has not been shown conclusively that mRNA deadenylation is the only downstream functional pathway of *Btg2* in cardiomyocytes since the previously determined molecular function of Btg2 and interacting effectors are wide-ranging, and EU-mediated RNA labeling could not distinguish mRNA from other non-coding RNAs. Nevertheless, the results of MS spectrometry analysis, regional quantitation of RNA amount in cardiomyocytes, and the specific suppression of cytosolic RNA abundance by Btg2 strongly support the functional relationship between Btg2 and the Ccr4–Not complex–mediated deadenylation system in cardiomyocytes.

Movement towards the anabolic state is an adaptive mechanism under stress conditions accompanied by increased levels of proteins and nucleic acids. The regulatory mechanisms that maintain the appropriate amount of these biomasses are critical components for cardiomyocyte function. Depleting the Ccr4–Not complex leads to heart failure with reduced systolic function in *Drosophila* and in mouse^57^, suggesting that the RNA-degrading mechanism is crucial and a regulatory factor like Btg2 is functionally important in cardiac pathophysiology. We speculate that *Btg2* must be strictly regulated as an immediate early and transiently expressed gene because of its strong anti-anabolic effect. Although mRNA deadenylation is a global mechanism, searching for the cardiomyocyte-specific target of *Btg2* is an attractive goal considering the role of RNA metabolism during the progression of pathological hypertrophy under clinical settings.

## Methods

### Reagents and antibodies

Phenylephrine (SIGMA), MG132 (SIGMA). Sarcomeric Alpha Actinin (EA-53, Abcam), Cardiac Troponin I (Abcam), Gapdh (clone 6C5, Millipore), Myc (D84C12, CST), RNA polymerase II (8WG16, Millipore), Btg2 (custom-made polyclonal antibody by immunizing rabbits, Cyclex), Cnot7 (18W, Santa-Cruz), α-Tubulin (MBL), anti-FLAG (M2, SIGMA), and Alexa 488-and Alexa 568-labeled secondary antibodies (Invitrogen).

### Cell Culture

Neonatal rat cardiomyocytes were prepared as previously described^58^. In brief, Harvested hearts were incubated in 0.25% trypsin/EDTA (Sigma) at 4 °C overnight and then digested with collagenase type II (Worthington). The cardiomyocyte fraction was collected after differential plating for 70 min at 37 °C, seeded and incubated with DMEM (Dulbecco’s Modified Eagle Medium, Gibco) containing 10% fetal bovine serum (FBS, Gibco) and penicillin, streptomycin and glutamine (Gibco), unless otherwise indicated. All procedures were performed in conformity with the *Guide for the Care and Use of Laboratory Animals published by the US National Institutes of Health* (NIH Publication, 8th Edition, 2011) and were approved by the Osaka University Committee for Laboratory Animal Use.

### Functional Enrichment

The gene lists with significant changes were subjected to gene ontology term analysis and functional enrichment clustering using the DAVID software (available at http://david.abcc.ncifcrf.gov/)^28^. Rattus norvegicus were selected as background species. The high-ranked functional terms defined by enrichment scores were listed.

### RNA extraction and quantitative real-time PCR

Total RNA was extracted using RNA-Bee reagent (Tel-Test) and converted to cDNA using high capacity RNA-to cDNA RT kit (Life technologies). Quantitative real-time PCR was performed with SYBR® green reagent or TaqMan® probe using StepOnePlus Real-Time PCR Systems (Applied Biosystems). All of the samples were processed in duplicate. The level of each transcript was quantified by the threshold cycle (Ct) method using Gapdh as an internal control.

### Transverse aortic constriction (TAC)

C57BL/6 mice aged 8 weeks and weighing 20–25 g were subjected to pressure overload, as described previously^59^. Briefly, the chest was entered via the second intercostal space at the upper left sternal border. After the arch of the aorta was isolated, a TAC was created using a 7-0 suture tied twice around a 30-gauge needle with the aortic arch between the innominate and left common carotid arteries. After the suture was tied, the needle was gently removed, yielding the constriction of the aorta.

### ChIP-sequencing, library preparation and data analysis

In ChIP experiments, isolated neonatal cardiomyocytes were cultured in medium containing 10 μM BrdU to inhibit proliferation of cardiac fibroblasts. Twenty-four hours after transduction of Ad-Myc or Ad-LacZ, cultured rat neonatal cardiomyocytes were washed by PBS, then cross-linked for 10 min at room temperature by addition of 16% paraformaldehyde to PBS at a final concentration of 0.5%. The crosslinking reaction was stopped by adding glycine to a final concentration of 175 mM. The fixed cells were washed with cold PBS on ice two times, harvested with cold PBS, then centrifuged. The cell pellet was flash frozen in liquid nitrogen and stored at −80 °C until following experiments. Forty μl Dynabeads^®^ Protein G (Life technology) were washed with PBS and incubated with 150 mM 1xRIPA buffer (50mM Tris-HCl [pH 8.0], 150 mM NaCl, 1 mM EDTA [pH 8.0], 0.1% SDS, 1% Triton X-100, and 0.1% Deoxycholic Acid) including 2 μg of the monoclonal anti-Myc antibody or monoclonal anti-RNA pol II antibody at 4 °C for 1 h. The crosslinked cell pellet was lysed with NP-40 buffer (10 mM Tris-HCl [pH 8.0], 10 mM NaCl, and 0.5% NP-40) and centrifuged. The cell pellet as nuclear fraction was lysed with SDS Lysis buffer (50 mM Tris-HCl [pH 8.0], 1% SDS, and 10 mM EDTA [pH8.0]), and sonicated by Bioruptor TOS-UCW-310-EX (Cosmo Bio Co., Ltd.). After centrifuge, the supernatant was collected as soluble chromatin fraction. The chromatin fraction was diluted two fold in ChIP dilution buffer (50 mM Tris-HCl [pH 8.0], 167 mM NaCl, 1.1% Triton X-100, and 0.11% Deoxycholic Acid Sodium Salt) and the 1.7% of the solution was stored as input control. The chromatin fraction was incubated with the antibody-conjugated beads at 4 °C with rotation overnight. The incubated beads were wash once with 150 mM 1xRIPA buffer, 500 mM 1xRIPA buffer, then two times with 1xTE buffer. The bound fraction and input were incubated with ChIP elution buffer (10 mM Tris-HCl [pH 8.0], 300 mM NaCl, 5 mM EDTA, and 0.5% SDS) and at 65 °C for 2 h. The eluted samples were incubated with RNase at 30 °C for 30 min, then incubated with Proteinase K at 55 °C for 1 h. The ChIP DNA and input were purified using QIAquick PCR purification Kit (QIAGEN) according to the manufacturer’s instructions and the DNA amount was quantified using Qubit® 2.0 fluorometer with Qubit® dsDNA HS Assay Kit (Life Technology). Each ChIP DNA and input samples were sheared by sonication, end-repaired, ligated to the sequencing adapters and amplified. The size distribution of library DNA was checked by Agilent 2100 Bioanalyzer, and ChIP efficiency was analyzed by quantitative PCR in each purification step. Sequencing of ChIP-Seq libraries were performed on a SOLiD instrument with 50-bp reads according to manufacturer’s instructions (Life Technologies). All experiments were performed on full sequencing slides with barcodes. Libraries were quantified by SYBR green quantitative-PCR (qPCR) to determine appropriate mixing ratios, which also depended on the desired sequencing depth for each of the libraries in the mixture. We repeated the two independent experiments for reproducibility.

Unfiltered sequence reads were aligned to the rat reference genome (rn4) using Bowtie^60^. The peak calling of Myc and pol II and the generation of WIG formatted files were performed using MACS (Model-based Analysis for ChIP-Seq)^24^ with the default parameters using ChIP sample and input control. Myc peaks at gene body including exonic, intronic, 5′ UTR, 3′ UTR and one thousand base pairs upstream were annotated using NCBI rn4 reference sequence. For the comparison of pol II peaks, we used a normalizing method by modifying the method previously described^15^. ChIP-seq reads aligning to the region were converted to WIG formatted files in which ChIP-seq values were numerically expressed in 10 bp bins. The density of reads in each region (represented as numerical values in the WIG files) was normalized to the total number of mapped reads to generate the read density per million mapped reads per bp^15^. Gene body defined from transcription start site to the end of transcript^26^ were divided into 10 blocks. At totally 25 blocks including upstream 5 blocks and downstream 10 blocks, the integrated values of pol II mapped reads were individually calculated from the WIG formatted files. Then, the quantified values were normalized by the length of gene body and the average density/bp value was calculated^25^. RNA pol II enrichment score at each gene was calculated by summing the normalized values of pol II peaks (density/bp) along with the gene body. All ChIP-seq data were deposited to GEO. The GEO accession number is GSE78934.

### Mass spectrometry analysis of the binding protein of Btg2

Cardiomyocytes were seeded at 1.5 × 10^6^ cells in 10 mm culture dishes and transduced with Ad-Btg2 or Ad-LacZ as a control. Forty-eight h after transduction, the cells were lysed with the lysis buffer (50 mM Tris–HCl (pH 7.4), 100 mM NaCl, 0.1 mM EDTA, 1% CHAPS, and protease inhibitor mixture (Nacalai Tesque)). The lysates were immunoprecipitated with anti-FLAG M2-agarose (Sigma-Aldrich) at 4 °C for 1 h. After extensive washing, the proteins were eluted with 250 μg/mL FLAG peptide at 4 °C for 30 min. The eluate was subjected to shotgun mass spectrometry analysis. The same eluate was electrophoresed on 4–12% NuPAGE Bis-Tris gel and silver-stained. Gel pieces, including specific bands from the silver-stained gels, were excised, digested with trypsin, and analyzed by mass spectrometry.

### EU Labeling of Cultured Cells

Cardiomyocytes (3 × 10^4 ^cells/well) were seeded on Greiner CELLSTAR® 96 well plates which were previously coated with collagen (Celmatrix Type I-C (KURABO)), and incubated in DMEM supplemented with 10% FBS. 1 mM EU was added to the complete culture medium from a 100 mM stock in DMSO. After EU labeling for the indicated time, cells were washed with PBS and fixed. The Labeled EU of the cells were visualized by Click-iT RNA imaging kits (Life Technology) according to the manufacturer’s instructions. The samples were incubated with the working solution of Click-iT reaction cocktail, containing the Alexa Fluor 594 azide and CuSO_4_, for 30 min at room temperature. We choose 594 probe to completely distinguish the fluorescent images of the labeled RNA from nuclear fluorescence stained by Hoechst. After removal of the reaction cocktail, cells were washed with Click-iT reaction rinse buffer and PBS, then transferred to immunostaining procedures. After incubation with blocking buffer (1% BSA in PBS), the cells were immunostained with anti-cardiac Troponin I polyclonal antibody for 1 h at room temperature. After washing with PBS, the cells were incubated with a goat anti–rabbit IgG secondary antibody, conjugated with Alexa Fluor 488 (Invitrogen) diluted by blocking buffer containing Hoechst. The immunofluorescent images were obtained using IN Cell Analyzer 6000. A total of 64 nonoverlap images (16 images per well) were obtained from each sample in one experiment using a 20 ×/0.45NA Nikon lens. The average data were obtained from at least three biological replicates. All the images are shown in figures after color conversion between green (488) and red (594). The obtained images were analyzed using IN Cell Developer toolbox (version1.9, GE) with the algorism described in Fig. S1.

## Additional Information

**How to cite this article**: Masumura, Y. *et al*. Btg2 is a Negative Regulator of Cardiomyocyte Hypertrophy through a Decrease in Cytosolic RNA. *Sci. Rep.* **6**, 28592; doi: 10.1038/srep28592 (2016).

## Supplementary Material

Supplementary Information
