# Supplementary material for: Btg2 is a Negative Regulator of Cardiomyocyte Hypertrophy through a Decrease in Cytosolic RNA
Source: Sci Rep. 2016 Jun 27;6:28592. doi: 10.1038/srep28592 (PMC4921833; doi:10.1038/srep28592)

Btg2 is a Negative Regulator of Cardiomyocyte Hypertrophy through a Decrease in Cytosolic RNA

Yuki Masumura[1], Shuichiro Higo[1], Yoshihiro Asano[1], Hisakazu Kato[2], Yi Yan[2], Saki Ishino[3], Osamu Tsukamoto[2], Hidetaka Kioka[1], Takaharu Hayashi[1], Yasunori Shintani[2], Satoru Yamazaki[4], Tetsuo Minamino[5], Masafumi Kitakaze[6], Issei Komuro[7], Seiji Takashima[2] and Yasushi Sakata[1]

[1]Department of Cardiovascular Medicine, [2]Department of Medical Biochemistry, Osaka University Graduate School of Medicine, Suita, Osaka 565-0871, Japan

[3]Center of Medical Innovation and Translational Research, Osaka University Graduate School of Medicine, Suita, Osaka 565-0871, Japan

[4]Department of Cell Biology and [6]Department of Clinical Medicine and Development, National Cerebral and Cardiovascular Center, Suita, Osaka 565-8565, Japan

[5]Department of Cardiorenal and Cerebrovascular Medicine, Faculty of Medicine, Kagawa University, 1750-1 Ikenobe, Miki-cho, Kita-gun, Kagawa 761-0793, JAPAN

[7]Department of Cardiovascular Medicine, Graduate School of Medicine, The University of Tokyo, Hongo, Bunkyo, Tokyo 113-8655, Japan

Correspondence should be addressed to Shuichiro Higo[a].

Assistant Professor, Department of Cardiovascular Medicine, Osaka University Graduate School of Medicine

Address: 2-2 Yamadaoka, Suita, Osaka 565-0871, Japan. Tel: +81-6-6879-3492, Fax: +81-6-6879-3493,

E-mail: higo-s@cardiology.med.osaka-u.ac.jp

Supplementary Materials and Methods

siRNA transfection and shRNA transduction.

siRNAs (50 nmol/l) were transfected into the precultured cardiomyocytes using Lipofectamine siRNA-max (Invitrogen) according to the manufacturer's instructions 4 to 6 h after primary culture. As a negative control, cells were transfected with siControl Non-Targeting siRNA (B-bridge).

For adenoviral shRNA construction, we used BLOCK-iT Adenoviral RNAi Expression System (Invitrogen). Oligonucleotides containing the target sequence were subcloned into pENTR-U6 vector and recombined into pAd/BLOCK-iT-DEST vector using LR clonase II (Invitrogen). Adenovirus encoding each shRNA were transduced into cardiomyocytes 4 to 6 h after primary culture at MOI 20. As a negative control, cells were transduced with shRNA against LacZ.

The used sequences are as follows.

siRNA against Rn Myc

sense: ccucagacaccgaggaaaaTT

antisense: uuuuccucggugucugaggTT

shRNA against Btg2 (#1), GGACGCACTGACCGATCATTA

shRNA against Btg2 (#2), GCAGTCCATCGAAGAACTACG

shRNA against LacZ, GCTACACAAATCAGCGATTT

shRNA against Cnot7, GCCTGAAGAAGAGCTTGATTT

PCR primers used for quantitative PCR.

Primers for SYBR Green-based qPCR

	Forward	Reverse
Rn Myc	TGAGCCCCTAGTGCTGCATG	TCTTGGCAGGGGGTTGCCTCT
Rn Npm1	TGAAGTGTGGTTCTGGGCCT	TACCACCTCCGGGAGCAGAT
Rn Gapdh	TCAACGGCACAGTCAAGG	CACGACATACTCAGCACC
Rn Btg1	CAAGTTCCTCCGCACCAA	CTGGGAACCAGTGATGTTTGT

Rn Btg2	CTCCAGGACGCACTGACCG	GGTCCATCTTGTGGTTGATGCGGA
Rn Btg3	TGATAAGGTGACCTCTGATTATCATT	TGGAAGAGGTGGGAATATCAGT
Rn Tob1	ACCAGCACTTTGAATGAGAATTT	TCAACTCCTCCACCAGAAAAA
Rn Tob2	GAAGCTGAGAGGGAGAGACCT	GCAGCAGGAAAGGAGAGTGA
Rn Cad	TGCCAGACCGTTGGGTACCCCT	GGTGCTCCTTAGAGACAGCAGCT
Rn Nppa	AACTGAGGGCTCTGCTCGCT	TACCGGAAGCTGTTGCAGCCTAGT
Rn Nppb	CAGTCTCCAGAACAATCCACGATGC	TGAGAGCTGTCTCTGAGCCA
Rn Acta1	CGAGCGTGGCTATTCCTTC	CCACGTAGCACAGCTTCTCTT
Rn Rpl3	TGACCGGCCAGGATCTAA	GTGGGGTTTCCACAATGG
Rn Rpl13	CTCGGCCGTTCTCCTGTA	GGTTGAACCACGTGTCCACT
Rn Gps2	ATACAAGCAGCCGGGAAGT	TTGGATGAAAGGGAGTCGAG
Rn Rab26	TCCTGGCTGGTACCTTCATC	GCCATCCACATCCAGAACTT
Rn B3galt4	ACAAGGTGGATCCTTGGAAA	TCCCATTCAGTCCTCTCACC
Rn Ndufb10	AAGCCTACCAGGACCGCTAC	CCTTGGCAGCCTTTCTCTC
Rn Rpl6	GGCAAGAGAGTGGTTTTCCTC	GGCGATGACAAACTTCTGGT
Rn Rpl12	GCAACGGTGCAACTTTCTTC	GGTGCACCTCAAGTACACGAC
Rn Rps18	CCACAGGAGGCCTACACG	CTGAAACTTCTCGGGGATCA
Rn Taf1d	CACAAATAGATGCAAAACAAATTAAAG	TCTTGCAACTGCTTGCTCA
Rn Cebpa	GCTGGTGATCAAGCAGGAG	GGGGCTGATAGGGGAAGAG
Rn Rps23	CGCAAAGGGAATTGTACTGG	GCACGAATGCTGTGATCTTC
Rn Rpl14	TGGTCGCAATCGTAGATGTT	GAGCTGCATGCATTTGAAAG
Rn Rpl38	CAAGGAGAAGGCGGAGAAG	TCACATGCAGAGGTCCTGTT
Rn Srm	GTCCTACGGGAAGTGGTGAA	GCCAGGCAGGAACTTCTTAG
Rn H2afj	GTGTCCTGCCCAACATCC	CTCAGCACCGGTAGAGTTCA
Rn Rpl8	TGCCTTCAGGGTCCAAGA	CCTTTAAGATAGGCTTGTCAATTCTG
Rn Rplp0	GATGCCCAGGGAAGACAG	CACAATGAAGCATTTTGGGTAG
Rn Rps19	CACGATGCCTGGAGTTACTG	TTTGGCCAACTTGACTGTGT

Rn Rps10	GGCAACGGAGTTCCAGTTTA	AGTTTATTCAACACAACATAATCTCCA
Rn Rps15a	GTGGCAGAACAATCTGCTCCCA	CCATGATGCCAGCCGAGGTCGTCA
Rn Eif5a	AGCACAGTAGCGGGTTCG	TTAAGAGGCTTCGATTCCAACT
Rn Rps14	CCAGAGGTGCAAGGAACTG	AGAGCTCTGAGGGCTGACTG
Rn Rpl9	TTGGATGGCATCTATGTTTCTG	TCTGAAGCTAGGAAACTGAGGTC
Rn Hspd1	ATGCTCACCGGAAGCCCTTGGT	TGTTGTCCCCAAACCCTGGAG
Rn Hist2h4	ACCCGTGGTGTACTGAAGGT	GCGTGCTCAGTGTAGGTGAC
Rn Rpl41	AGAATGCGCAGGCTGAAG	CAGCAGCATACAGTCCAACC
Rn Hist1h2b1	CCGGATACCGGAATCTCTTC	GCGCTCGAAGATGTCGTT
Rn Hspa8	TCCTCACTATCGAGGATGGAA	AAAGTCTTCTCCGCCCAAGT
Rn Rps27	CAGCCTACAGGTGGAAAAGC	CTTGAATCAGGGGCTTTCAG

TaqMan Gene Expression Assay

Mm Myc	Mm00487804_m1		
Mm Nppa	Mm01255747_g1	Mm Btg2	Mm00476162_m1

TaqMan Rodent GAPDH Control Reagents

Primers for SYBR Green-based ChIP-qPCR

Rn Btg2 -26	AAAGCGCAGCCCGGGGAAAGT	CGTGGCTCATACCGGTGGTTGA
Rn Btg2 +53	TCAACCACCGGTATGAGCCACG	CGAGTCCTCAGGAGACTGGT
Rn Btg2 +2657	AGGTGGCTCAAAGCTCCAGG	CTCCAGGACCCAACCGCAGGAA

Animals

All procedures were performed in conformity with the *Guide for the Care and Use of Laboratory Animals published by the US National Institutes of Health* (NIH Publication, 8th Edition, 2011) and were approved by the Osaka University Committee for Laboratory Animal Use. Anesthesia and postoperative analgesia were performed with medetomidine (0.3 mg/kg), midazolam (4 mg/kg) and butorphanol (5 mg/kg) via intraperitoneal administration.

Adequate anesthesia was ensured by monitoring the respiration rate and absence of a response to a paw pinch. Euthanasia when collecting heart tissues after TAC was performed by overdose of the mixed anesthesia via intraperitoneal administration.

Statistical analyses

Our data are expressed as means ± S.D. of at least three independent experiments, unless otherwise indicated. The two-tailed Student's *t*-test or one-way ANOVA with repeated measures were used to analyze differences between two groups. p-value < 0.05 was considered statistically significant.

Constructs.

The coding sequence of mouse Myc gene (NM_001177354) was amplified by PCR from mouse heart cDNA library and subcloned into pENTR/D-TOPO (Invitrogen) (pENTR-Myc) using Gateway Technology. The deletion mutants of DNA binding domain(ΔC) were generated by PCR using pENTR-Myc as a template. The coding sequence of mouse Btg2 gene (NM_007570) was amplified by PCR from mouse heart cDNA library and subcloned into pENTR/D-TOPO, then recombined into pEF-DEST51 vector containing FLAG sequence, generating C-terminally FLAG tagged Btg2 (Btg2-Flag WT). D105A mutation was generated by PCR with the primers targeting the sites of substitution using Btg2-FLAG as a template. For adenoviral construction and viral production, we used ViraPower Adenoviral Expression System (Invitrogen) for overexpression.

Western Blotting

For western blotting, cells were washed with cold PBS and lysed with SDS buffer (50mM Tris-HCl (pH 7.4), 10% SDS, 5 mM EDTA) to inhibit proteinases during cell lysis. The protein concentration was determined by BCA Protein Assay Kit (Thermo). Proteins were separated by SDS-PAGE and transferred to PVDF membrane. Anti-Myc antibody was diluted at 2 μg/mL by 3% nonfat milk. Anti-Btg2 antibody was diluted at 2μg/mL by Can Get Signal Immunoreaction Enhancer Solution (Toyobo). After blocking with 3% nonfat milk for 1 h, the transferred membrane was incubated with primary antibody at 4 °C overnight and with secondary antibody at room temperature for 30 min. The membrane signals were detected by chemiluminescence using ECL or ECL prime reagent (GE). Gapdh or

Tubulin was used as control for equal loading and transfer. The intensity of the protein band was quantified by ImageQuantTL (GE).

Supplementary Figure S1

(A) Twenty-four hours after transduction with Ad-LacZ, Ad-Myc, or Ad-MycΔC at MOI 20 in cardiomyocytes, *Npm1* mRNA levels were determined by quantitative real-time PCR, normalized to *Gapdh* mRNA levels (n=3, mean±SD). *: $p < 0.05$ vs Ad-LacZ.

(B) Rat neonatal cardiomyocytes were treated with 1 mM EU for 1 h before being fixed and immunostained. Nuclear DNA was stained with Hoechst, and cardiomyocytes were highlighted specifically by Troponin I immunostaining. Scale bar: 10 μm.

(C) Adenovirally transduced LacZ, Myc, or MycΔC in cardiomyocytes at increasing MOI were detected by western blotting. Gapdh protein was used as a loading control. Before harvesting, cells were treated with 5 μM MG132 for 1 h.

(D) Analytical procedures for the quantitative evaluation of RNA amount or cardiomyocyte surface area using IN Cell Developer Toolbox (GE). Each italic phrase indicates the command operation defined in the software. Scale bar: 50 μm.

<u>Nucleus Recognition (1), (2)</u>

I. Select the channel with the image of the nuclei marker (Hoechst) (1).

II. Select *Segmentation* option (greater than 65 μm^2) most appropriate for an accurate nuclear segmentation.

III. Use *Postprocessing* operations (*Erosion*, *Watershed clump breaking* and *Sieve)* to refine nuclear (2).

<u>Cardiomyocyte Recognition (3), (4)</u>

I. Select the channel with the image of the cardiomyocyte marker (Troponin I) (3).

II. Select *Segmentation* option (intensity segmentation) and set an intensity threshold that identify whole cells (cell bodies).

III. Use *Postprocessing Sieve* operation (greater than 300 μm^2) to filter out debris, *Clump Breaking* operation using ‘Nuclei’ as ‘seed’ (4). For quantitative evaluation, we used *Border Object Removal* to omit cardiomyocytes that extend over the edge of the image.

IV. Create a linked *One to One* target set to link ‘Nucleus’ (primary target set) with ‘Cardiomyocyte’ (secondary target set), to count the number of cardiomyocytes. Set *Overlap* conditions that more than 80% primary target within secondary target.

EU-labeled RNA Recognition (5), (6)

I. Select the channel with the image of the labeled RNA (EU) (5).

II. Select ***Segmentation*** option (intensity segmentation) and set an intensity threshold that will identify EU staining in cells (6).

Cardiomyocyte-specific Nuclear EU-labeled RNA Recognition

To extract target set 'RNA' contained only in nuclei of cardiomyocyte from 'RNA' contained in whole cells, we created a Macro that includes the following operation.

I. *Combine-Min* with image (2) and image (4) to select the nuclei of cardiomyocyte (7).

II. *Combine-Min* with image (4) and image (6) to select only the target set 'RNA' included in cardiomyocyte (8).

III. *Combine-Min* with image (7) from image (8) to select target set 'RNA' included in nuclei of cardiomyocyte (9).

Cardiomyocyte-specific Cytosolic EU-labeled RNA Recognition

To extract target set 'RNA' contained only in cytosol of cardiomyocyte from 'RNA' contained in whole cells, we created a Macro that includes the following operation.

I. *Combine-Min* with image (2) and image (4) to select the nuclei of cardiomyocyte (7).

II. *Combine-Min* with image (4) and image (6) to select only the target set 'RNA' included in cardiomyocyte (8).

III. *Subtraction* of image (7) from image (8) to select target set 'RNA' included in cytosol of cardiomyocyte (10).

Generate *User Defined Measurement* to report

I. To calculate the mean cell surface area of cardiomyocytes
[Area<\Hoechst_TnI\TnI>] and Select ***Statistical function - Mean*** for this measure.

II. To calculate EU intensity per cell
[DxA<Sum:\EU>]/[Count<Sum:\Hoechst_TnI\TnI>] and Select ***Statistical function - Mean*** for this measure.

III. Finally subtracted the quantitative measurements of the intensity of the background image to correct background fluorescence.

(E) The representative images of the quantified nuclear (upper) or cytosolic (lower) RNA amount in individual cardiomyocytes defined by *One to One target link*. The cell number was determined by Nuclei count.

Supplementary Figure S2

(A) Outline of the quantification algorithm used to analyze RNA Pol II recruitment profiles for relative comparison. The gene body was defined to extend from the transcription start site to the end of each transcript, and was divided into 10 blocks. At a total of 25 blocks, including 5 upstream blocks and 10 downstream blocks, the integrated values of Pol II mapped reads represented as WIG formatted files were calculated individually. The quantified values were subsequently normalized by the gene length.

(B) Myc and pol II peaks at the representative genes in cardiomyocytes transduced with adenovirus encoding LacZ or Myc in the replicated ChIP experiments. Myc-targeted genes with pol II recruitment in cardiomyocytes were functionally involved in ribosomal biogenesis, nucleolus function or translation. Vertical axis indicates the density of reads normalized to the total number of mapped reads producing read density per million mapped reads per bp.

(C) Average Pol II occupancy along the gene body in the replicated experiment (ChIP 2) at all 16,313 rat RefSeq genes (top), at the 3,080 genes preferentially bound by Myc (middle), or at the remaining 13,233 genes not bound by Myc (bottom).

Supplementary Figure S3

(A) Myc-targeted genes without pol II recruitment in cardiomyocytes. Myc bound the genes encoding cardiac specific transcription factors, structural proteins and natriuretic peptides, but did not induce pol II recruitment at these genes. Vertical axis indicates the read density per million mapped reads per bp.

(B) Twenty-four hours after transduction with Ad-LacZ or Ad-Myc at MOI 20 in cardiomyocytes cultured under 0% or 10% serum condition, the mRNA expression levels of the indicated genes were determined by quantitative real-time PCR, normalized to *Gapdh* mRNA levels (n=3, mean±SD). *: $p < 0.01$, #: $p < 0.05$ vs Ad-LacZ.

Supplementary Figure S4

(A) The quantified RNA Pol II enrichment score (upper: ChIP1, lower: ChIP2) of the top 25 ranked genes according to the ratio of the enrichment score (Ad-Myc/Ad-LacZ).

(B) Twenty-four hours after transduction with Ad-LacZ or Ad-Myc at MOI 20 in cardiomyocytes, the mRNA expression levels of the 25 high-ranked genes listed in (A) were evaluated by quantitative real-time PCR, normalized to *Gapdh* mRNA levels (n=3 to 4, mean±SD). *: $p < 0.01$, #: $p < 0.05$ vs Ad-LacZ.

(C) The ratio of the RNA pol II enrichment score (Ad-Myc/Ad-LacZ) of the genes listed in Fig. 3A..

(D) The mRNA expression levels of the genes listed in Fig. 3A were evaluated by quantitative real-time PCR, normalized to *Gapdh* mRNA levels (n=3, mean±SD). *: $p < 0.01$, #: $p < 0.05$ vs Ad-LacZ.

Supplementary Figure S5

(A) Cardiomyocytes seeded in 96 well plates were starved for 12 h and treated with PE for 12 h before they were fixed and immunostained. Fluorescence images were obtained with an imaging cytometer. The representative images acquired in the quantification algorithm to calculate individual cell surface area in cardiomyocytes are shown (details are shown in Fig. S1D). Scale bar: 50 μm.

(B) Cardiomyocytes seeded in 96 well plates were transduced with shRNA against either *LacZ* or *Btg2*. Twenty-four hours after transduction, cardiomyocytes were starved for 12 h and treated with PE for 12 h before they were fixed and immunostained. Fluorescence images were obtained using an imaging cytometer. Scale bar: 50 μm.

(C) Cardiomyocytes were transduced with Ad-Btg2 or LacZ at MOI 20. Twenty-four hours after transduction, the cells

were fixed and immunostained. Btg2-FLAG expression was detected by anti-FLAG antibody. Scale bar: 50 μm.

(D) The result of mass spectrometry analysis of the binding proteins of the overexpressed Btg2 in cardiomyocytes. The individual bands only observed in the lane of Btg2-bound proteins were excised, in-gel digested by trypsin, then subjected to mass spectrometry analysis. The identified proteins are listed in the right table. The components of Ccr4-Not complex are highlighted in yellow.

(E) Levels of wild-type Btg2-FLAG and $Btg2^{D105A}$-FLAG at increasing MOI expressed in cardiomyocytes were detected by western blotting using anti FLAG antibody. Tubulin was used as a loading control.

(F) Cardiomyocytes were co-transduced with Ad-Myc and either Ad-LacZ, Ad-Btg2 WT, or Ad-$Btg2^{D105A}$. Twenty-four hours after transduction, the average cardiomyocyte surface areas were calculated and normalized by Ad-LacZ control (n=3, mean±SD). *: p<0.05 vs Ad-LacZ.

(G) Cardiomyocytes were transduced with shRNA against either *LacZ* or *Cnot7*. Forty-eight hours after transduction, *Cnot7* mRNA levels were determined by quantitative real-time PCR, normalized to *Gapdh* mRNA levels (n=3, mean±SD). *: p < 0.05 vs LacZ.

(H) Cardiomyocytes were either transduced with shRNA against LacZ or Cnot7. Twelve hours after initial transduction, Ad-LacZ or Ad-Btg2 WT were transduced. Twenty-four hours after transduction, cells were labeled with EU for 24 h before they were fixed and immunostained. Cytosolic EU intensity was quantified (n=3, mean±SD). *: p < 0.05 vs Ad-LacZ. #: p < 0.05 vs shRNA against LacZ.

Table S1

RNA pol II enrichment score at the genes in which pol II recruitment exhibited more than two fold increase in the cardiomyocytes transduced with Ad-Myc than that with Ad-LacZ control.

Table S2

The result of DAVID functional annotation clustering analysis of the Myc bound genes. The detailed annotation terms and categories of the genes with (944 genes) or without (2136 genes) pol II enrichment are shown.

Table S3

The result of the mass spectrometry analysis of the binding protein eluted from the immunoprecipitated sample of FLAG-tagged Btg2.

Supplementary_Figure_1

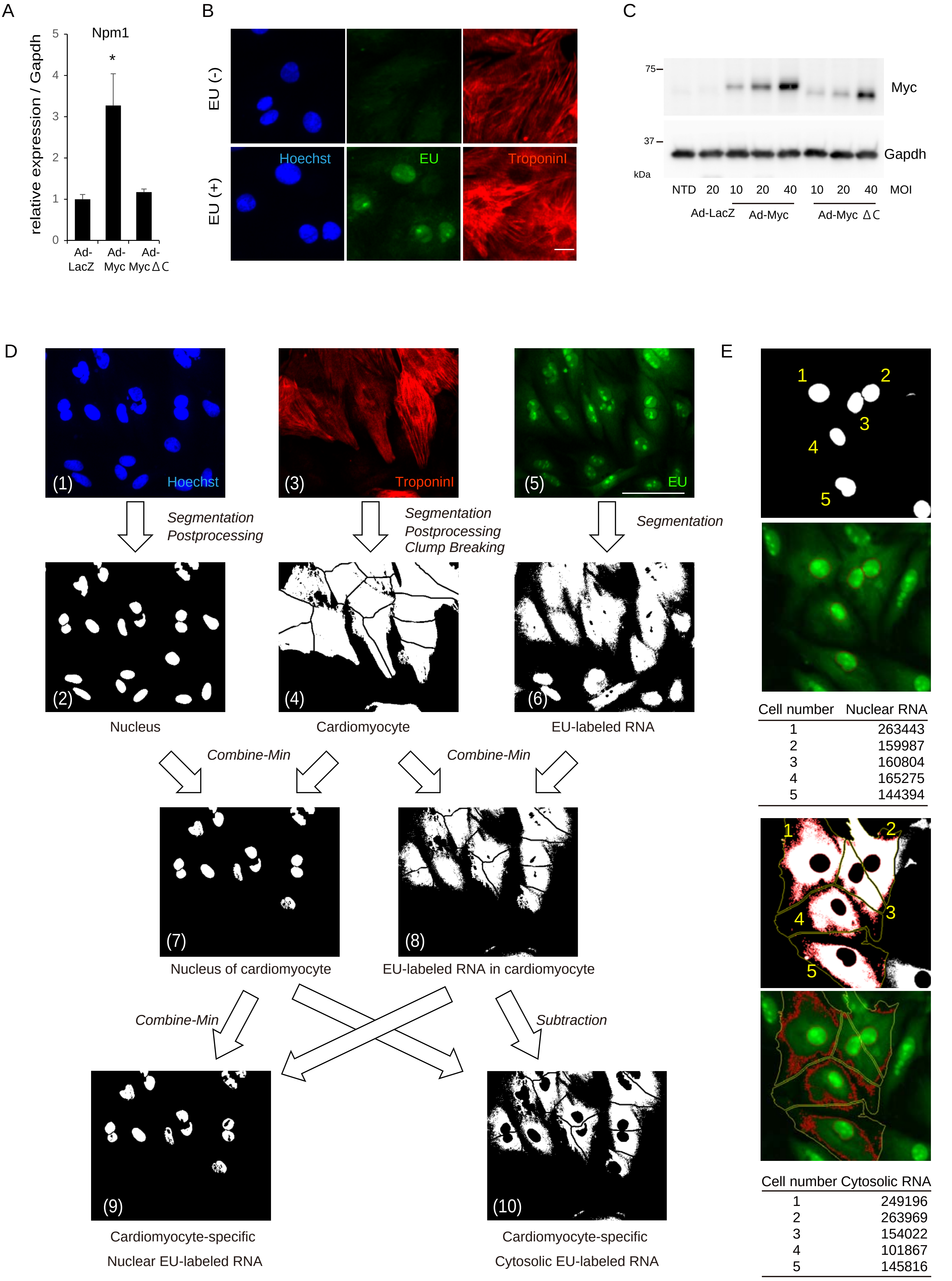

Cell number	Nuclear RNA
1	263443
2	159987
3	160804
4	165275
5	144394

Cell number	Cytosolic RNA
1	249196
2	263969
3	154022
4	101867
5	145816

Supplementary_Figure_2

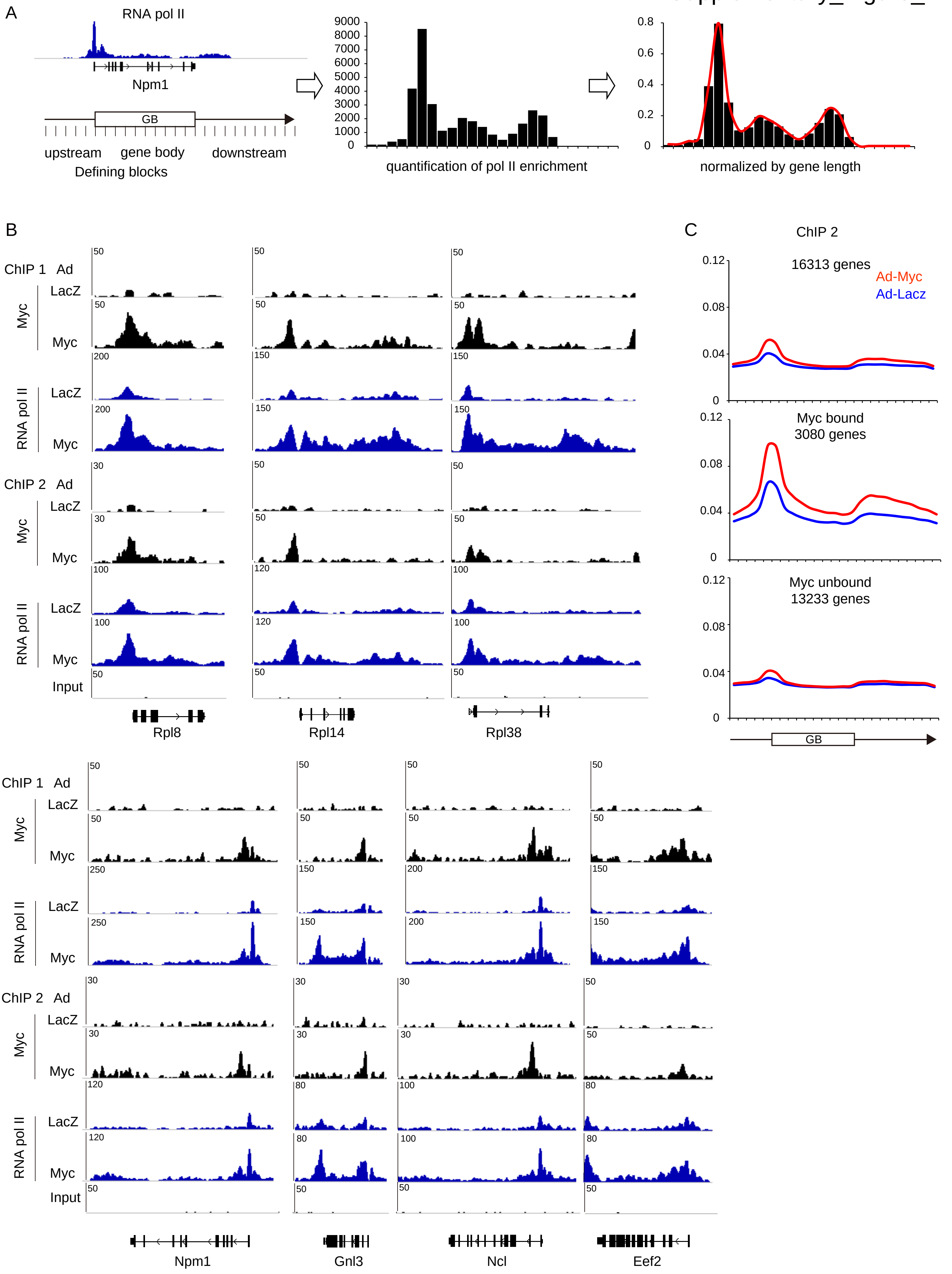

Supplementary_Figure_3

A

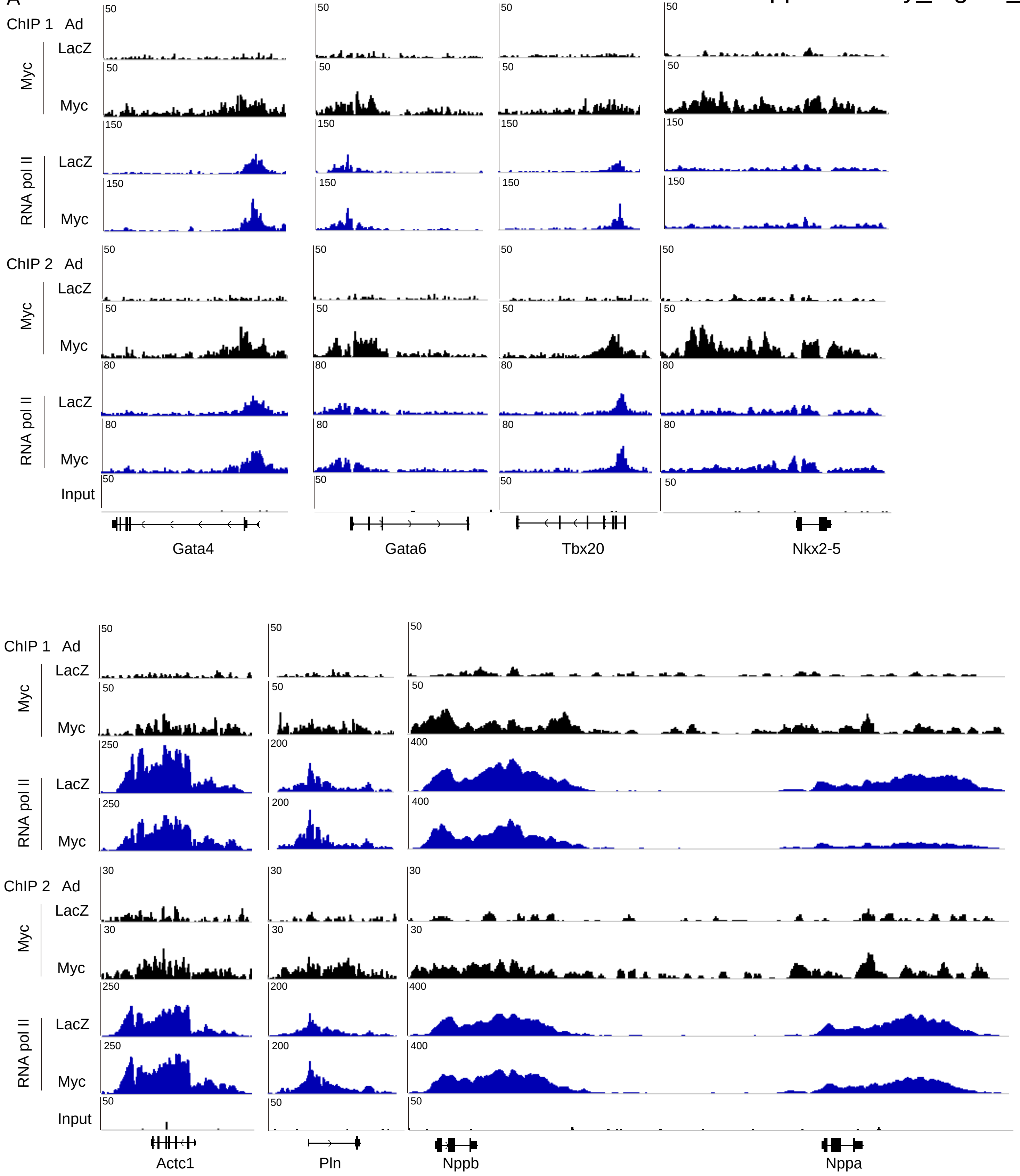

B

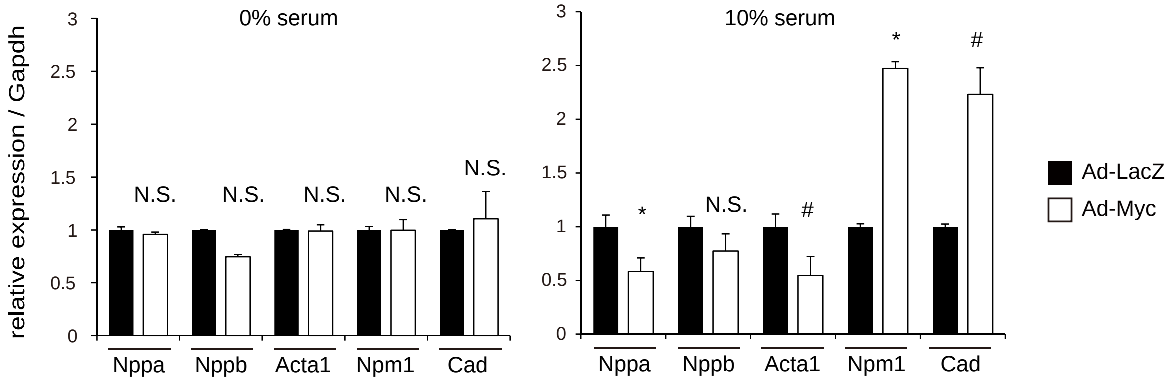

Supplementary_Figure_4

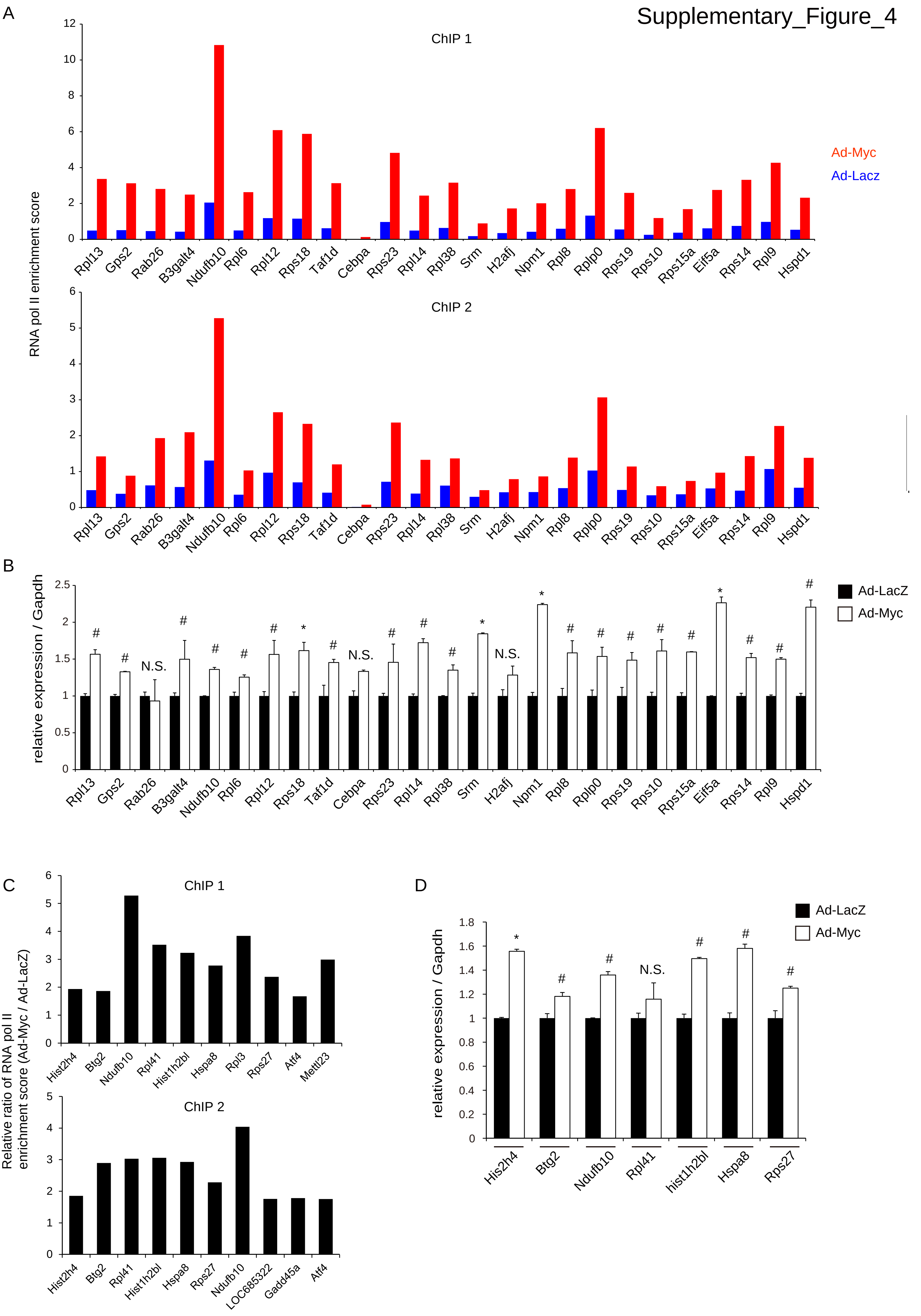

Supplementary_Figure_5

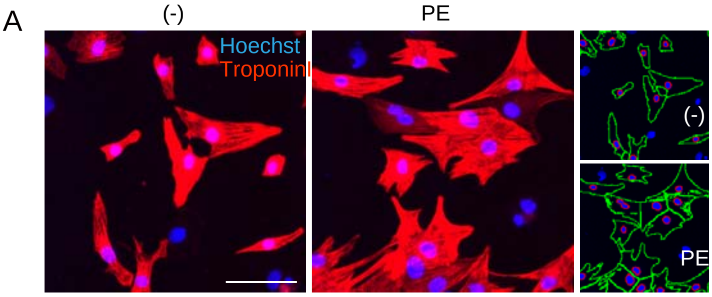

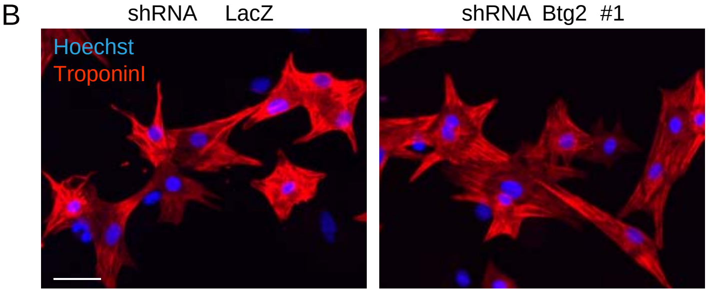

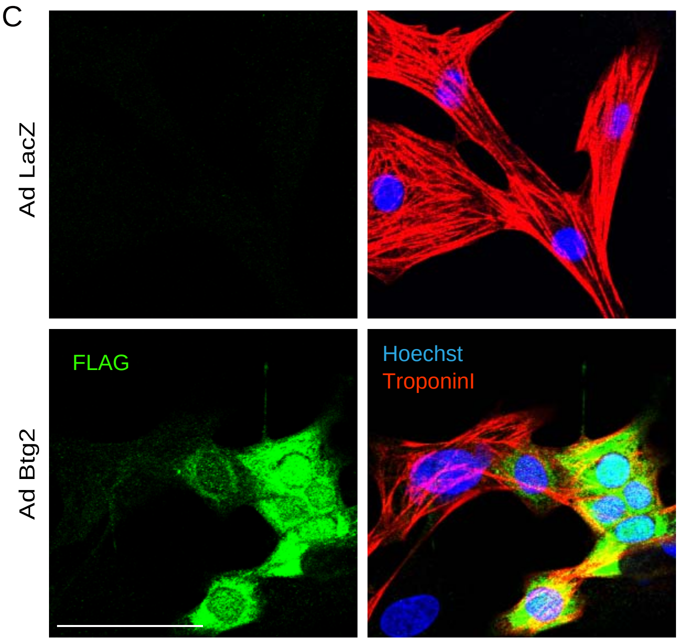

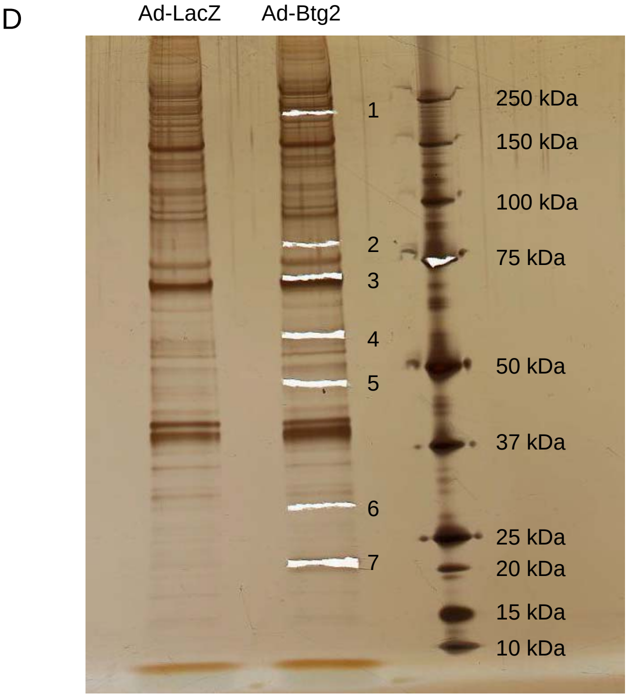

Identified Proteins Band 1	Molecular Weight
Cnot1 CCR4-NOT transcription complex subunit 1	267 kDa
Pi4ka Uncharacterized protein	232 kDa
**Identified Proteins Band 2**	
Cnot10 CCR4-NOT transcription complex subunit 10	82 kDa
**Identified Proteins Band 3**	
Hspa5 78 kDa glucose-regulated protein	72 kDa
Prmt5 protein arginine N-methyltransferase 5	73 kDa
Hspa8 Heat shock cognate 71 kDa protein	71 kDa
Hspa9 Stress-70 protein, mitochondrial	74 kDa
Ddx3x Uncharacterized protein	73 kDa
Pabpc1 Polyadenylate-binding protein 1	71 kDa
**Identified Proteins Band 4**	
Psmd3 Uncharacterized protein	61 kDa
Stk38l Serine/threonine kinase 38-like	54 kDa
Hnrnpk Uncharacterized protein	51 kDa
Cnot2 Uncharacterized protein (Fragment)	59 kDa
Cnot6l CCR4-NOT transcription complex subunit 6-like	62 kDa
Cnot6 CCR4-NOT transcription complex subunit 6	63 kDa
Cct8 Uncharacterized protein	60 kDa
**Identified Proteins Band 5**	
RGD1560909 UPF0760 protein C2orf29 homolog	55 kDa
Eef1a1 Elongation factor 1-alpha 1	50 kDa
Dctn2 Dynactin subunit 2	44 kDa
Tuba1b Uncharacterized protein	50 kDa
Rpl4 60S ribosomal protein L4	47 kDa
Psmc4 26S protease regulatory subunit 6B	47 kDa
**Identified Proteins Band 6**	
Rps3 40S ribosomal protein S3	27 kDa
Rqcd1 Cell differentiation protein RCD1 homolog	34 kDa
Rps4x;LOC100362640;LOC100364620 40S ribosomal protein S4, X isoform	30 kDa
Rpl8;LOC100360117 60S ribosomal protein L8	28 kDa
Cnot7 Uncharacterized protein	33 kDa
Slc25a5 ADP/ATP translocase 2	33 kDa
**Identified Proteins Band 7**	
Rpl23a 60S ribosomal protein L23a	18 kDa
Psmb5 Proteasome subunit beta type-5	29 kDa
LOC497813;Rps7 40S ribosomal protein S7	22 kDa
Rpl11 60S ribosomal protein L11	20 kDa
Rpl18a 60S ribosomal protein L18a	21 kDa
Cryab Alpha-crystallin B chain	20 kDa
Rps5 Uncharacterized protein	23 kDa
Rpl24 60S ribosomal protein L24	18 kDa
Btg2 Protein BTG2	18 kDa
RGD1561815 Uncharacterized protein	19 kDa
LOC100364558;LOC100360846 proteasome subunit beta type 6-like	25 kDa
Psmb2 Proteasome subunit beta type-2	23 kDa
Psmb3 Proteasome subunit beta type-3	23 kDa

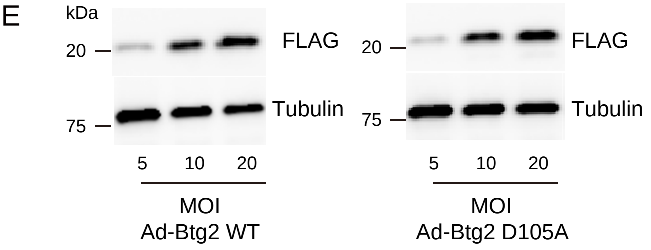

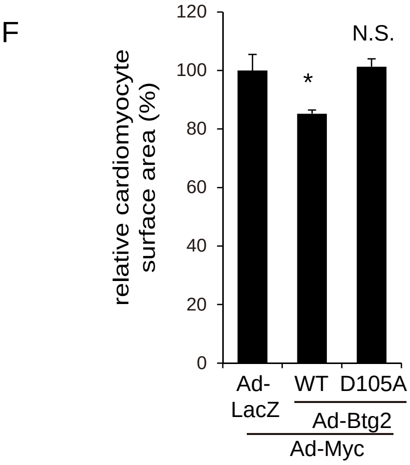

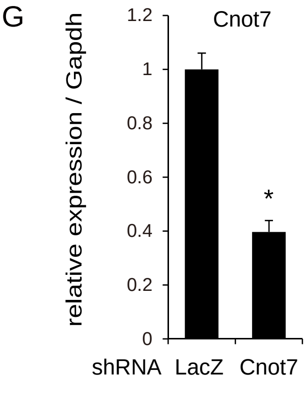

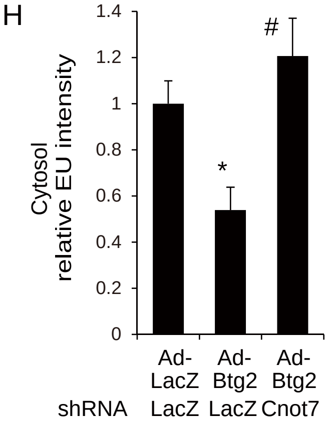

Supplement: Supplementary Information [file srep28592-s1.pdf]
